# A fractional system of delay differential equation with nonsingular kernels in modeling hand-foot-mouth disease

**DOI:** 10.1186/s13662-020-02993-3

**Published:** 2020-09-29

**Authors:** Behzad Ghanbari

**Affiliations:** 1 Department of Engineering Science, Kermanshah University of Technology, Kermanshah, Iran; 2 Department of Mathematics, Faculty of Engineering and Natural Sciences, Bahçeşehir University, 34349 Istanbul, Turkey

**Keywords:** Mathematical modeling of infectious diseases, The Atangana–Baleanu fractional derivative, Approximate solutions, Predictor–corrector scheme, Fractional delay differential equations

## Abstract

In this article, we examine a computational model to explore the prevalence of a viral infectious disease, namely hand-foot-mouth disease, which is more common in infants and children. The structure of this model consists of six sub-populations along with two delay parameters. Besides, by taking advantage of the Atangana–Baleanu fractional derivative, the ability of the model to justify different situations for the system has been improved. Discussions about the existence of the solution and its uniqueness are also included in the article. Subsequently, an effective numerical scheme has been employed to obtain several meaningful approximate solutions in various scenarios imposed on the problem. The sensitivity analysis of some existing parameters in the model has also been investigated through several numerical simulations. One of the advantages of the fractional derivative used in the model is the use of the concept of memory in maintaining the substantial properties of the understudied phenomena from the origin of time to the desired time. It seems that the tools used in this model are very powerful and can effectively simulate the expected theoretical conditions in the problem, and can also be recommended in modeling other computational models in infectious diseases.

## Introduction

Infectious diseases are a branch of science that deals with the diagnosis and treatment of diseases caused by microorganisms. While many infectious diseases such as tuberculosis, plague, leprosy, smallpox, and the flu have existed throughout the history of the world, humans have been fighting microorganisms for centuries. Although some infectious diseases, such as smallpox, have been eradicated through vaccination, new infectious diseases have emerged, such as AIDS and COVID-19. With globalization, global warming, and increasing travel, whether newly emerging viral diseases or resistant bacterial infections, infectious diseases remain at the forefront.

The hand-foot-mouth disease (HFMD) is one of the most common infectious diseases that affect many people around the world. The disease is caused by some viruses like Coxsackievirus A16 and Enterovirus 71. The disease is more common in children under 5 years old. More precisely, the prevalence of the disease is much more common in children under three years of age. Besides, there have been numerous cases of the disease in adults around the world. In most cases, patients have mild symptoms for 7 to 10 days [1]. Some of these symptoms are fever and flu-like symptoms, mouth sores, and skin rash. So far, two main ways of transmitting this contagious disease have been identified: the first way is transmitted through contact with an infected person or contact with tools used by infected people. The disease can also be spread through coughing or sneezing in the air and transmitted through the respiratory transmission to another person.

The first cases of HFMD cases were clinically reported in Canada and New Zealand in 1957. The disease has been reported in many parts of the world, including some parts of Asia, Europe, and the United States. The disease first appeared in Shanghai, China, and spread rapidly to other parts of the country, including Beijing, Shandong, and Jiangxi provinces. According to our information, the epidemic of this disease occurs in cycles of two or three years. The best time to peak the spread of the disease is usually in the summer. Heat and humidity are known as two main factors that aggravate the spread of the disease. The first case of the disease was seen in Thailand in 2003. In a short time, signs of this contagious disease were visible in all cities and provinces of this country [32]. The first national study describing a large number of deaths caused in this country by the disease during an outbreak in 2011 was presented in [46]. HFMD has also been found in many Indian major states [38].

Unfortunately, no specific curative treatment has been found for this viral disease. However, several simple preventive measures such as avoiding direct contact with infected people, quarantining infected children at school, cleaning common utensils regularly, and disinfecting contaminated surfaces have been confirmed as the most effective ways to reduce transmission of the virus to other people in the community.

Due to the high prevalence of this disease in different parts of the world and the importance of identifying and controlling the factors affecting its prevalence, many extensive kinds of research have been conducted by researchers from various scientific aspects. In [26], a new method for the disease prediction using GeoDetector and a Long Short-Term Memory neural network (LSTM) has been proposed. In [40], the authors applied optimal control theory to the HFMD model, including the treatment and vaccination interventions. They presented some control strategies based on minimizing the cost of the intervention and minimizing the number of infected people. Very recently, a fractional-order model has been utilized to describe the transmission of HFMD in [39]. In this paper, the authors considered two major cases of constant and optimal control. In each case, the existence and uniqueness of positive solutions and the sufficient conditions for the existence and stability of equilibriums were investigated. It is important to mention that their study on the optimality control conditions is based on Pontryagin’s maximum principle. Another interesting study on the disease was conducted to investigate the role of air pollution in the prevalence and incidence of the disease in the warm and cold seasons in Wuhan, China [47]. In [42], the authors investigated some strategies to control the disease using a system of ordinary differential with two delay parameters. To estimate and better understand the transmissibility of HFMD, a susceptible–infected–recovered (SIR) model has been utilized in [26]. They claim that the main reason for this choice is that the incubation period of the disease is less than one week. In [13], another new SIR model has been developed to fit the surveillance data containing valuable information on the severity of HFMD in order to accurately estimate the basic reproductive number ($R_{0}$) of the disease. In [27], a simple SEIR model has been examined to investigate the dynamics of the disease among young children. A discrete SEIADR (susceptible–exposed–infectious–asymptomatic–dead–recovered) epidemic model has been also developed in [34]. To read more articles, please refer to [12, 16, 17, 28, 31, 33, 45, 50, 51].

Fractional differential calculus has been used in modeling many phenomena in everyday real-world applications [18, 20–23, 25]. Therefore, due to the importance and scope of applications, many efficient numerical methods specific to each of these types of fractional operators have also been introduced [3–8, 10, 11, 15, 19, 24, 30, 35, 37, 43]. However, in [41] the author has conducted a review to point some possible problems and difficulties arising in the construction of fractional-dynamic model analogs of standard models by using fractional calculus. This stems from the fact that some fundamental properties of fractional derivatives (such as the multiplicity principle, the solvability and correspondence principles, and the interpretability principle) violate various known standard rules and properties that are fulfilled for derivatives of integer order.

Information retention (or so-called memory effect) is one of the most basic properties of fractional-order derivatives. This significant property has made these operators one of the most efficient tools in computational models arising in biology. In other words, the basic information of the model from the beginning of time is utilized to characterize the behavior of the phenomenon at any desired time. The use of fractional derivatives has made it possible to employ such a valuable feature. To take advantage of memory-related benefits in the context of fractional-order derivatives, our main objective is to study a nonlinear system of the delayed-fractional model in studying HFMD. For this purpose, the present contribution is structured as follows: Some mathematical background, mainly about recent definitions on fractional calculus model, is formulated in Sect. 2. The proposed model is introduced in Sect. 3. In Sect. 4, we investigate some mathematical frameworks of the model, including the equilibrium points, the basic reproduction number, and the existence and uniqueness of the model’s solution. Then, we present some corresponding numerical simulations in Sect. 5. Finally, some conclusions are drawn in the last section of the article.

## Some basic preliminaries on fractional operators

Employing new definitions in mathematics always makes extensive progress in modeling various phenomena around us in the world. One of these new and powerful topics is the use of fractional calculus concepts. In this section, we will have an overview of some of known basic definitions corresponding to fractional calculus that are used in what follows. For this purpose, we present the definition of Riemann–Liouville fractional integral and derivative operators ${} ^{\mathsf{RL}} \mathcal{I}^{ \alpha }$ and ${} ^{\mathsf{RL}} \mathcal{D}^{ \alpha }$ of order $\alpha >0$, respectively. Then, we define the Caputo fractional derivative ${} ^{\mathsf{C}} \mathcal{D}^{ \alpha }$ of order $\alpha >0$ as a modification of the Riemann–Liouville fractional derivative. Further, we present some properties of the Mittag-Leffler function $E_{\alpha ,\beta }(z)$, $\alpha ,\beta >0$ (see [48]).

### Definition 1

For a given integrable function $\mathcal{S}(t)$, the fractional integral operator in the Riemann–Liouville sense ${} ^{\mathsf{C}} \mathcal{D}^{ -\alpha }$ of order $\alpha >0$ is given by [36]

1 $$ {} ^{\mathsf{C}} \mathcal{I}^{ \alpha } \mathcal{S}(t) = \frac{1}{\Gamma (\alpha )} \int _{0}^{t} (t-\varsigma )^{\alpha -1} \mathcal{S}(\varsigma )\,d\varsigma , $$

where $\Gamma (\cdot)$ denotes the well-known gamma function.

### Definition 2

For a given function $\mathcal{S}(t)$ in $C[0,T]$, the fractional derivative operator in the Riemann–Liouville sense ${} ^{\mathsf{C}} \mathcal{I}^{ -\alpha }$ of order $\alpha >0$ is given by

$$ {} ^{\mathsf{C}} \mathcal{D}^{ \alpha } \mathcal{S}(t)=\textstyle\begin{cases} \frac{1}{\Gamma (n-q)} \frac{d^{n}}{dt^{n}} \int _{0}^{t} \frac{\mathcal{S}(\varsigma )}{(t-\varsigma )^{\alpha -n+1}}\,d\varsigma ,\quad n-1\leq \alpha < n, n\in \mathbb{N}, \\ \frac{d^{n}}{dt^{n}} \mathcal{S}(t), \quad \alpha =n, n \in \mathbb{N}. \end{cases} $$

### Definition 3

Let $T>0$ and $\mathcal{S}(t)\in C^{n}[0,T]$. The Caputo fractional derivative operator ${} ^{\mathsf{C}} \mathcal{D}^{ \alpha }$ of order $\alpha >0$ is defined by

$$ {} ^{\mathsf{C}} \mathcal{D}^{ \alpha } \mathcal{S}(t)=\textstyle\begin{cases} \frac{1}{\Gamma (n-q)} \int _{0}^{t} \frac{\mathcal{S}^{(n)}(\varsigma )}{(t-\varsigma )^{\alpha -n+1}}\,d\varsigma , \quad n-1< \alpha < n, n\in \mathbb{N}, \\ \frac{d^{n}}{dt^{n}} \mathcal{S}(t),\quad q=n, n\in \mathbb{N}. \end{cases} $$

The following two important propositions are consequences of the above definitions:

$$ {} ^{\mathsf{C}} \mathcal{D}^{ \alpha } \bigl( {} ^{\mathsf{C}} \mathcal{I}^{ \alpha } \mathcal{S}(t) \bigr) =\mathcal{S}(t),\qquad {} ^{\mathsf{C}} \mathcal{I}^{ \alpha } \bigl( {} ^{\mathsf{C}} \mathcal{D}^{ \alpha }\mathcal{S}(t) \bigr)=\mathcal{S}(t)-\sum _{k=0}^{n-1} \frac{h^{(k)}(0)}{k!}t^{k},\quad t>0. $$

Definitions (2) and (3) are different from each other, and the relation between the two types of fractional derivatives is as follows:

$$ {} ^{\mathsf{C}} \mathcal{D}^{ \alpha }\mathcal{S}(t)={}^{RL}_{0} \mathscr{D}_{t}^{\alpha }\mathcal{S}(t)-\sum _{k=0}^{n-1} r_{k}^{ \alpha }(t) \mathcal{S}^{(k)}(0),\quad r_{k}^{\alpha }(t)= \frac{t^{k-\alpha }}{\Gamma (k+1-\alpha )}. $$

The Caputo derivative has the main advantage that the initial condition of the corresponding problem has the same value as the ordinary differential equation. Moreover, for a constant-valued function, the Caputo derivative is zero.

### Definition 4

The Mittag-Leffler function of two parameters is given by

2 $$ \mathcal{E}_{\alpha ,\beta }(z)=\sum_{k=0}^{\infty } \frac{z^{k}}{\Gamma (\alpha k+\beta )},\quad z\in \mathbb{C}, $$

where $\alpha ,\beta >0$, $\mathbb{C}$ denotes the complex plane.

For $\alpha =\beta =1$, the Mittag-Leffler function $\mathcal{E}_{1,1}(z)$ gives the exponential function $\exp (z)$. Moreover, the Mittag-Leffler function satisfies the following useful equality:

$$ \mathcal{E}_{\alpha ,\beta }(z) =z \mathcal{E}_{\alpha ,\alpha +\beta }(z)+ \frac{1}{\Gamma (\beta )},\quad \alpha ,\beta >0. $$

### Lemma 1

*For* $a\in \mathbb{R}$*and* $\alpha ,\beta >0$, *we obtain*

3 $$ \mathcal{L}\bigl(t^{\alpha -1}\mathcal{E}_{\alpha ,\beta }(at)\bigr)= \frac{s^{\alpha -\beta }}{s^{\alpha }-a}. $$

*Also*,

4 $$ \mathcal{L}\bigl( {} ^{\mathsf{C}} \mathcal{D}^{ \alpha }\mathcal{S}(t) \bigr) =s^{ \alpha -1} \widehat{\mathcal{S}}(s) -\sum _{k=0}^{n-1}\mathcal{S}^{(k)}(0) s^{\alpha -k-1}, $$

*where* $\widehat{\mathcal{S}}(s)=\mathcal{L}(\mathcal{S}(t))$.

### Definition 5

The AB-Caputo derivative operator ${} ^{\mathsf{AB}} \mathscr{D}^{ \alpha }$ of $\mathcal{S}(t)$ is defined as follows [9]:

5 $$ {} ^{\mathsf{AB}} \mathcal{D}^{ \alpha } \mathcal{S}(t) = \frac{{ {\mathsf{AB}} (\alpha )}}{{1 - \alpha }} \int _{0}^{t} \mathcal{E}_{\alpha ,1 } \biggl[ {-\frac{\alpha }{1 - \alpha }(t - \varsigma ) } \biggr]\dot{\mathcal{S}}( \varsigma )\,d\varsigma , \quad \alpha \in (0,1) $$

and ${\mathsf{AB}} (\alpha )=1-\alpha +\alpha / \Gamma (\alpha )$.

### Definition 6

The AB-Caputo integral operator of order *α* is defined as

6 $$\begin{aligned} {} ^{\mathsf{AB}} \mathcal{I}^{ \alpha } \mathcal{S}(t) &= \frac{1-\alpha }{{\mathsf{AB}} (\alpha )} \mathcal{S} (t )+ \frac{\alpha }{\Gamma (\alpha ){\mathsf{AB}} (\alpha )} \int _{0}^{t} \mathcal{S}(\varsigma ) (t- \varsigma )^{\alpha -1}\,d\varsigma ,\quad 0< \alpha \leq 1. \end{aligned}$$

### Lemma 2

*For the operators defined in Equations* (6) *and* (5), *the following union is established* [2]:

7 $$\begin{aligned} {} ^{\mathsf{AB}} \mathcal{I}^{ \alpha } \bigl({} ^{\mathsf{AB}} \mathcal{D}^{ \alpha } \mathcal{S}(t) \bigr) = \mathcal{S}(t)- \mathcal{S}(0). \end{aligned}$$

## The delayed-fractional version of the model

In recent years, modeling real-world problems using the fractional delay differential equations (FDDEs) has attracted much attention among mathematicians, physicists, and engineers. Due to the wide application of these equations, the theoretical and practical aspects of this category of equations have been extensively studied by researchers in many research articles such as [42, 44, 49].

In the light of these facts and after employing the AB-Caputo fractional derivative ${} ^{\mathsf{AB}} \mathcal{D}^{ \alpha }$ and defining two-time delays $\tau _{1}$ and $\tau _{2}$ in the model presented in [42], we arrive at the following FDDE for the spread of the disease:

8 $$\begin{aligned} \begin{aligned} &{} ^{\mathsf{AB}} \mathcal{D}^{ \alpha } \mathcal{S}(t) = (1-\rho ) b \mathcal{N}(t)-\lambda \mathcal{S}(t)-(1-\epsilon ) \lambda \mathcal{S}(t-\tau _{1})-\rho _{1} \mathcal{S}(t-\tau _{1}) \\ &\hphantom{{} ^{\mathsf{AB}} \mathcal{D}^{ \alpha } \mathcal{S}(t) =}{}-\bigl(\gamma \mathcal{N}(t) +\mu +\rho _{2} \bigr) \mathcal{S}(t)+w \mathcal{R}(t), \\ &{} ^{\mathsf{AB}} \mathcal{D}^{ \alpha }\mathcal{T}_{\nu }(t) = \rho _{1} \mathcal{S}(t-\tau _{1})+\epsilon \mathcal{V}_{\nu }(t)-\bigl(\mathcal{N}(t) \gamma +\eta _{4}+\mu \bigr) \mathcal{T}_{\nu }(t), \\ &{} ^{\mathsf{AB}} \mathcal{D}^{ \alpha } \mathcal{I}_{c}(t) = \lambda \mathcal{S}(t)+(1-\epsilon ) \lambda \mathcal{S}(t-\tau _{1})-\bigl( \gamma \mathcal{N}(t) +\eta _{2}+\delta + \mu \bigr) \mathcal{I}_{c}(t), \\ &{} ^{\mathsf{AB}} \mathcal{D}^{ \alpha }\mathcal{R} (t) =\eta _{2} \mathcal{I}_{c}(t)-\bigl(\gamma \mathcal{N}(t) + \mu +\omega \bigr) \mathcal{R}(t)+ \eta _{3} \mathcal{V}_{ca}(t)+ \eta _{4} \mathcal{T}_{\nu }(t), \\ &{} ^{\mathsf{AB}} \mathcal{D}^{ \alpha }\mathcal{V}_{\nu }(t) =b\rho \mathcal{N}(t)+\rho _{2} \mathcal{S}(t)-\phi \lambda \mathcal{V}_{\nu }(t)-\bigl( \gamma \mathcal{N}(t) +\epsilon +\mu \bigr) \mathcal{V}_{\nu }(t), \\ &{} ^{\mathsf{AB}} \mathcal{D}^{ \alpha }\mathcal{V}_{ca}(t) =\phi \lambda \mathcal{V}_{\nu }(t)-\bigl(\gamma \mathcal{N}(t) +\eta _{3}+ \delta +\mu \bigr) \mathcal{V}_{ca}(t), \end{aligned} \end{aligned}$$

where $\lambda = \beta (\mathcal{I}_{c}(t-\tau _{2}) +\mu \mathcal{V}_{ca}(t- \tau _{2}))/\mathcal{N}(t)$, and subject to given initial conditions

$$\begin{aligned}& \mathcal{S}(t) = \mathcal{\psi }_{1} (t), \qquad \mathcal{T}_{\nu }(t) = \mathcal{\psi }_{2} (t),\qquad \mathcal{I}_{c}(t) = \mathcal{\psi }_{3} (t),\qquad \mathcal{R}(t) = \mathcal{\psi }_{4} (t), \\& \mathcal{V}_{\nu }(t) = \mathcal{\psi }_{5} (t), \qquad \mathcal{V}_{ca}(t) = \mathcal{\psi }_{6} (t),\quad t\in [-\tau , 0], \tau =\max \{\tau _{1}, \tau _{2}\}. \end{aligned}$$

The effective populations in this model are categorized into six state variables as follows: the susceptible subpopulation $\mathcal{S}(t)$, the treated and vaccinated subpopulation $\mathcal{T}_{\nu }(t)$, the clinically infectious subpopulation $\mathcal{I}_{c}(t)$, the recovered subpopulation $\mathcal{R}(t) $, the vaccinated subpopulation $\mathcal{V}_{\nu }(t) $, and finally, the vaccinated carrier subpopulation $\mathcal{V}_{ca}(t)$. We symbolize the sum of all subpopulations by $\mathcal{N}(t)$. In this model, the transmission rate is denoted by *β*. Moreover, *b* is the birth rate, $\eta _{2}$ represents the transforming rate from $\mathcal{I}_{c}(t) $ to $\mathcal{R}(t)$. Also, $\eta _{2}$ is utilized to describe the vaccinated carrier rate, *ω* is the recovery rate, *ϕ* is the rate of protection loss, $\tau _{1}$ and $\tau _{2}$ are two delay parameters. Moreover, *μ* denotes the natural death rate, treating and vaccinating rate of susceptible people is shown by $\rho _{1}$, culling of clinical infective, and vaccinated carrier rate is denoted by *δ*. Finally, the rates corresponding to the treating vaccinated, vaccinating susceptible, and recovery of treated people are *ϵ*, $\rho _{2}$, and $\eta _{4}$, respectively [29].

In consecutive subsections, we analyze some theoretical aspects of the fractional model outlined in (8).

### The equilibrium points

The equilibrium points of the system are determined as follows:

- The disease-free equilibrium of the model is $\mathcal{B}_{1}= (\mathcal{S}_{1},\mathcal{T}_{\nu 1},0, \mathcal{R}_{1}, \mathcal{V}_{\nu 1},0 )$, where
  9 $$\begin{aligned}& \begin{aligned} &\mathcal{S}_{1}= \frac{ ((1-\rho ) (b^{3}+ ( \omega +\epsilon +\eta _{4} ) b^{2}+ ((\omega +\eta _{4})b\epsilon +\omega \eta _{4} )b )+\epsilon \omega \eta _{4} ) r}{\gamma M_{1}}, \\ &\mathcal{T}_{\nu 1}= \frac{(b+\omega )r (\epsilon (b\rho +\rho _{1}+\rho _{2} )+b\rho _{1}(1-\rho ) )}{\gamma M_{1}}, \\ &\mathcal{R}_{ 1}= \frac{(b+\omega )r (\epsilon (b\rho +\rho _{1}+\rho _{2} )+b\rho _{1}(1-\rho ) )}{\gamma M_{1}}, \\ &\mathcal{V}_{ \nu 1}= \frac{rb (b+\omega +\eta _{4} ) \rho \rho _{1}+r(b+\eta _{4})(b+\omega ) (b\rho +\rho _{2} ) }{\gamma M_{1}}, \end{aligned} \end{aligned}$$
  and
  $$\begin{aligned} M_{1} =& b^{3} + (\omega + \epsilon +\rho _{1} + \rho _{2} + \eta _{4})b2 \\ &{} + \bigl(( \omega + \rho _{1} + \rho _{1} + \eta _{4}) + (\omega + \rho _{1}+ \rho _{2})\eta _{4} + \omega (\rho _{1}+\rho _{2})\bigr)b \\ &{}+ \bigl((\omega + \rho _{1}+\rho _{2})\bigr)\eta _{4} + \omega (\rho _{1}+\rho _{2}) + \omega \eta _{4}\rho _{2}. \end{aligned}$$
- The endemic equilibrium of the model is $\mathcal{B}_{2}= (\mathcal{S}_{2},\mathcal{T}_{\nu 2}, \mathcal{I}_{c 2},\mathcal{R}_{2}, \mathcal{V}_{\nu 2},\mathcal{V}_{ca} )$, where
  10 $$\begin{aligned}& \mathcal{S}_{1}= \frac{ (1-\rho )b(r/\gamma )+\omega \mathcal{R}_{1}}{(2-\epsilon )\lambda '+b+\rho _{1}+\rho _{2}}, \\& \mathcal{T}_{\nu 1}= \frac{\rho _{1} ( (1-\rho )b b(r/\gamma ) +\omega \mathcal{R}_{1} )}{A_{4}}+ \frac{\epsilon b(r/\gamma ) b p}{ (b+\mu _{4} ) (\lambda '\phi +b+\epsilon )} \\& \hphantom{\mathcal{T}_{\nu 1}=}{}+ \frac{\epsilon \rho _{2} ( (1-\rho )b b(r/\gamma )+\omega \mathcal{R}_{1} )}{ (\lambda '\phi +b+\epsilon ) A_{4}}, \\& \begin{aligned} &\mathcal{I}_{c 1}= \frac{ (2-\epsilon )\lambda ' ( (1-\rho )b b(r/\gamma )+\omega \mathcal{R}_{1} )}{ (\delta +b+\eta _{2} ) ( (2-\epsilon )\lambda '+b+\rho _{1}+\rho _{2} )} , \\ &\mathcal{R}_{ 1}=\frac{1}{1-A_{2}} \biggl( \frac{A_{3}}{(b+\omega )(\delta +b+\eta _{2})A_{4}}\end{aligned} \\& \hphantom{\mathcal{R}_{ 1}=}{}+A_{5} \biggl( \frac{\rho }{(b+\eta _{4})(\delta +b+\eta _{3})}+ \frac{\rho _{2}(1-\rho )}{A_{4} (\delta +b+\eta _{3})} \biggr) \biggr), \\& \mathcal{V}_{ \nu 1}= \frac{b(r/\gamma )b\rho }{\lambda '\phi +b+\epsilon }+ \frac{ \rho _{2} ((1-\rho )bb(r/\gamma )+\omega \mathcal{R}_{ 1} ) }{ ({\lambda '\phi +b+\epsilon } ) ((2-\epsilon )\lambda '+b+\rho _{1}+\rho _{2} )}, \\& \mathcal{V}_{ ca 1}=\frac{\lambda ' \phi }{\delta +b+\eta _{3}} \biggl( \frac{b(r/\gamma )b\rho }{\lambda '\phi +b+\epsilon }+ \frac{ \rho _{2} ((1-\rho )bb(r/\gamma )+\omega \mathcal{R}_{ 1} ) }{ ({\lambda '\phi +b+\epsilon } ) ((2-\epsilon )\lambda '+b+\rho _{1}+\rho _{2} )} \biggr), \end{aligned}$$
  and
  11 $$\begin{aligned}& A_{1}={ ({b+\omega } ) \bigl((2- \epsilon )\lambda '+b+ \rho _{1}+\rho _{2} \bigr)}, \\& A_{2}=\frac{\omega }{A_{1}} \biggl( \frac{\eta _{2} (2-\epsilon )\lambda ' }{\delta +b+\eta _{2}} \\& \hphantom{A_{2}=}{}+ \frac{ \eta _{4} (\lambda ' \phi \rho _{1}+(b+\epsilon )\rho _{1}+\epsilon \rho _{2} ) }{ ({\lambda '\phi +b+\epsilon } ) (b+\eta _{4} )}+ \frac{ \eta _{3} \lambda ' \phi \rho _{2} }{ ({\lambda '\phi +b+\epsilon } ) (\delta +b+\eta _{3} )} \biggr), \\& A_{3}=(1-\rho )b K \bigl((2-\epsilon )\lambda '(b+ \eta _{4})\eta _{2}+ \eta _{4}\rho _{1}(\delta +b+\eta _{2}) \bigr), \\& A_{4}= \bigl((2-\epsilon )\lambda '+b+\rho _{1}+\rho _{2} \bigr) (b+ \eta _{2}), \\& A_{5}= \frac{ b b(r/\gamma ) (\eta _{3} \lambda ' \phi (b+\eta _{4})+\epsilon \eta _{4}(\delta +b+\eta _{3}) ) }{ ({\lambda '\phi +b+\epsilon } ) (b+\omega )}, \\& A_{6}= \bigl((b+\epsilon ) (b+\rho _{1}) (b\delta + \eta _{3}) (b\delta + \eta _{2}) \bigr), \end{aligned}$$

A detailed survey of the stability of these equilibrium points can be found in the reference [42].

### The basic reproduction number

Employing the next generation matrix method [14], the basic reproduction number of model (8) is given by [42]

12 $$ R_{0}= \frac{\beta \phi \mu \rho b }{ (\epsilon +b ) (\eta _{3}+b+\delta )}+ \frac{\beta (2-\epsilon )(1-\rho )b}{(b+\rho _{1})(b+\delta +\eta _{2})}. $$

### Existence of the solution

To investigate and prove the existence of a solution for the model, we can first apply the AB-Caputo integral operator (6) to the sides of model (8) to give the following equalities:

13 $$\begin{aligned}& \mathcal{S} (t)- \mathcal{S} (0)= \frac{1-\alpha }{ \mathsf{AB} (\alpha )} \mathcal{N}_{1}\bigl( { \mathcal{ \textbf{F(t)}}} \bigr)+ \frac{\alpha }{ \mathsf{AB} (\alpha )\Gamma (\alpha )} \int _{0}^{t} (t- \varsigma )^{\alpha -1} \mathcal{N}_{1}\bigl( \textbf{F} ( \varsigma )\bigr)\,d\varsigma , \\& \mathcal{T}_{\nu }(t) - \mathcal{T}_{\nu }(0) = \frac{1-\alpha }{ \mathsf{AB} (\alpha )} \mathcal{N}_{2}\bigl( { \mathcal{ \textbf{F(t)}}} \bigr) + \frac{\alpha }{ \mathsf{AB} (\alpha )\Gamma (\alpha )} \int _{0}^{t} (t- \varsigma )^{\alpha -1} \mathcal{N}_{2}\bigl( \textbf{F} ( \varsigma )\bigr)\,d\varsigma , \\& \begin{aligned} &\mathcal{I}_{c}(t) - \mathcal{I}_{c}(0)= \frac{1-\alpha }{ \mathsf{AB} (\alpha )} \mathcal{N}_{3}\bigl( { \mathcal{ \textbf{F(t)}}} \bigr) + \frac{\alpha }{ \mathsf{AB} (\alpha )\Gamma (\alpha )} \int _{0}^{t} (t- \alpha )^{\alpha -1} \mathcal{N}_{3}\bigl( \textbf{F} ( \varsigma )\bigr)\,d\varsigma , \\ &\mathcal{R} (t) - \mathcal{R}(0)= \frac{1-\alpha }{ \mathsf{AB} (\alpha )} \mathcal{N}_{4} \bigl( { \mathcal{\textbf{F(t)}}} \bigr) + \frac{\alpha }{ \mathsf{AB} (\alpha )\Gamma (\alpha )} \int _{0}^{t} (t- \alpha )^{\alpha -1} \mathcal{N}_{4}\bigl( \textbf{F} ( \varsigma )\bigr)\,d\varsigma ,\end{aligned} \\& \mathcal{V}_{\nu }(t) - \mathcal{V}_{\nu }(0)= \frac{1-\alpha }{ \mathsf{AB} (\alpha )} \mathcal{N}_{5}\bigl( { \mathcal{ \textbf{F(t)}}} \bigr) + \frac{\alpha }{ \mathsf{AB} (\alpha )\Gamma (\alpha )} \int _{0}^{t} (t- \alpha )^{\alpha -1} \mathcal{N}_{5}\bigl( \textbf{F} ( \varsigma )\bigr)\,d\varsigma , \\& \mathcal{V}_{ca}(t) - \mathcal{V}_{ca}(0)= \frac{1-\alpha }{ \mathsf{AB} (\alpha )} \mathcal{N}_{6}\bigl( { \mathcal{ \textbf{F(t)}}} \bigr) + \frac{\alpha }{ \mathsf{AB} (\alpha )\Gamma (\alpha )} \int _{0}^{t} (t- \alpha )^{\alpha -1} \mathcal{N}_{6}\bigl( \textbf{F} ( \varsigma )\bigr)\,d\varsigma , \end{aligned}$$

where $\textbf{F}(t)= [\mathcal{S}(t),\mathcal{T}_{\nu }(t), \mathcal{I}_{c}(t),\mathcal{R}(t),\mathcal{V}_{\nu }(t),\mathcal{V}_{ca}(t) ] $, and

14 $$\begin{aligned} \begin{aligned} &\mathcal{N}_{1}\bigl( \textbf{F}(t) \bigr)= (1-\rho ) b \mathcal{N}(t)-\lambda \mathcal{S}(t)-(1- \epsilon ) \lambda \mathcal{S}(t )-\rho _{1} \mathcal{S}(t ) \\ &\hphantom{\mathcal{N}_{1}\bigl( \textbf{F}(t) \bigr)=}{}-\bigl( \gamma \mathcal{N}(t) + \mu +\rho _{2}\bigr) \mathcal{S}(t)+w \mathcal{R}(t), \\ &\mathcal{N}_{2}\bigl( \textbf{F}(t) \bigr)= \rho _{1} \mathcal{S}(t )+\epsilon \mathcal{V}_{\nu }(t)-\bigl( \mathcal{N}(t) \gamma +\eta _{4}+ \mu \bigr) \mathcal{T}_{\nu }(t), \\ &\mathcal{N}_{3}\bigl( \textbf{F}(t)\bigr)= \lambda \mathcal{S}(t)+(1- \epsilon ) \lambda \mathcal{S}(t )-\bigl(\gamma \mathcal{N}(t) +\eta _{2}+ \delta +\mu \bigr) \mathcal{I}_{c}(t), \\ &\mathcal{N}_{4}\bigl( \textbf{F}(t)\bigr)= \eta _{2} \mathcal{I}_{c}(t)-\bigl( \gamma \mathcal{N}(t) + \mu +\omega \bigr) \mathcal{R}(t)+\eta _{3} \mathcal{V}_{ca}(t)+ \eta _{4} \mathcal{T}_{\nu }(t), \\ &\mathcal{N}_{5}\bigl( \textbf{F}(t)\bigr)= b\rho \mathcal{N}(t)+ \rho _{2} \mathcal{S}(t)-\phi \lambda \mathcal{V}_{\nu }(t)-\bigl(\gamma \mathcal{N}(t) +\epsilon +\mu \bigr) \mathcal{V}_{\nu }(t), \\ &\mathcal{N}_{6}\bigl( \textbf{F}(t)\bigr)= \phi \lambda \mathcal{V}_{\nu }(t)-\bigl(\gamma \mathcal{N}(t) +\eta _{3}+\delta +\mu \bigr) \mathcal{V}_{ca}(t). \end{aligned} \end{aligned}$$

Defining $\textbf{N}( \textbf{F}(t))= [ \mathcal{N}_{1}( \textbf{F}(t)), \mathcal{N}_{2}( \textbf{F}(t)),\ldots , \mathcal{N}_{6}( \textbf{F}(t)) ] $, and $\textbf{F}_{0}= [\mathcal{S}(0),\mathcal{T}_{\nu }(0), \mathcal{I}_{c}(0),\mathcal{R}(0), \mathcal{V}_{\nu }(0),\mathcal{V}_{ca}(0) ] $, Equation (13) can be considered as follows:

15 $$ \textbf{F}(t)- {\textbf{F}_{0}}= \frac{1-\alpha }{ \mathsf{AB} (\alpha )} \textbf{N}\bigl( \textbf{F}(t)\bigr)+ \frac{\alpha }{ \mathsf{AB} (\alpha )\Gamma (\alpha )} \int _{0}^{t} (t- \varsigma )^{\alpha -1} \textbf{N}\bigl( \textbf{F} {( \varsigma )}\bigr)\,d\varsigma . $$

The following iterative process can be defined:

16 $$ \begin{aligned}& {\textbf{F}}_{n}(t)- {\textbf{F}_{0}}= \frac{1-\alpha }{ \mathsf{AB} (\alpha )} \textbf{N}\bigl( { \textbf{F}}_{n-1}(t) \bigr)+ \frac{\alpha }{ \mathsf{AB} (\alpha )\Gamma (\alpha )} \int _{0}^{t} (t- \varsigma )^{\alpha -1} \textbf{N}\bigl( {\textbf{F}}_{n-1} {( \varsigma )}\bigr)\,d\varsigma , \\ &{\textbf{F}}_{0}(t)= {\textbf{F}_{0}}. \end{aligned}$$

Subtracting two consecutive terms gives

17 $$\begin{aligned}& {\textbf{F}}_{n}(t)- {\textbf{F}}_{n-1}(t) \\& \quad = \frac{1-\alpha }{ \mathsf{AB} (\alpha )} \bigl[ \textbf{N}\bigl( {\textbf{F}}_{n-1}(t) \bigr)- \textbf{N}\bigl( {\textbf{F}}_{n-2}(t)\bigr) \bigr] \\& \qquad {}+ \frac{\alpha }{ \mathsf{AB} (\alpha )\Gamma (\alpha )} \int _{0}^{t} (t- \varsigma )^{\alpha -1} \bigl[\textbf{N}\bigl( {\textbf{F}}_{n-1} {(\varsigma )} \bigr)-\textbf{N}\bigl( {\textbf{F}}_{n-2} {( \varsigma )} \bigr) \bigr]\,d\varsigma . \end{aligned}$$

For simplicity, we set $\Xi _{n}(t)={\textbf{F}}_{n}(t)- {\textbf{F}}_{n-1}(t)$. Hence, one has

18 $$ {\textbf{F}}_{n}(t)=\sum _{i=0}^{n} {\Xi }_{i}(t). $$

Thus, we get

19 $$\begin{aligned}& \bigl\Vert {\Xi }_{n}(t) \bigr\Vert = \bigl\Vert {\textbf{F}}_{n}(t)- { \textbf{F}}_{n-1}(t) \bigr\Vert , \\& \bigl\Vert {\Xi }_{n}(t) \bigr\Vert = \biggl\Vert \frac{1-\alpha }{ \mathsf{AB} (\alpha )} \bigl[ \textbf{N}\bigl( {\textbf{F}}_{n-1}(t) \bigr)- \textbf{N}\bigl( {\textbf{F}}_{n-2}(t)\bigr) \bigr] \\& \hphantom{\bigl\Vert {\Xi }_{n}(t) \bigr\Vert =}{}+ \frac{\alpha }{ \mathsf{AB} (\alpha )\Gamma (\alpha )} \int _{0}^{t} (t- \varsigma )^{\alpha -1} \bigl[\textbf{N}\bigl( {\textbf{F}}_{n-1} {(\varsigma )} \bigr)-\textbf{N}\bigl( {\textbf{F}}_{n-2} {( \varsigma )} \bigr) \bigr]\,d\varsigma \biggr\Vert . \end{aligned}$$

Hence

$$\begin{aligned} \bigl\Vert {\Xi }_{n}(t) \bigr\Vert \leq& \frac{1-\alpha }{ \mathsf{AB} (\alpha )}\Vert \textbf{N}\bigl( { \textbf{F}}_{n-1}(t) \bigr)- \textbf{N}\bigl( {\textbf{F}}_{n-2}(t)\bigr) \biggl\Vert \\ &{} +\frac{\alpha }{ \mathsf{AB} (\alpha )\Gamma (\alpha )} \int _{0}^{t} (t-\varsigma )^{\alpha -1} \biggr\Vert \textbf{N}\bigl( {\textbf{F}}_{n-1} {( \varsigma )}\bigr)-\textbf{N}\bigl( {\textbf{F}}_{n-2} {( \varsigma )}\bigr)\Vert \,d\varsigma . \end{aligned}$$

Whenever **N** satisfies the Lipschitz condition with respect to **F**, thus

$$\begin{aligned} \bigl\Vert {\Xi }_{n}(t) \bigr\Vert \leq& \frac{1-\alpha }{ \mathsf{AB} (\alpha )}\textbf{L} \bigl\Vert {\textbf{F}}_{n-1}(t)- { \textbf{F}}_{n-2}(t) \bigr\Vert \\ &{}+ \frac{\alpha \textbf{L}}{ \mathsf{AB} (\alpha )\Gamma (\alpha )} \int _{0}^{t} (t-\varsigma )^{\alpha -1} \bigl\Vert {\textbf{F}}_{n-1}(t)- {\textbf{F}}_{n-2}(t) \bigr\Vert \,d\varsigma . \end{aligned}$$

Hence the following inequality will be obtained:

$$\begin{aligned} \bigl\Vert {\Xi }_{n}(t) \bigr\Vert \leq \frac{1-\alpha }{ \mathsf{AB} (\alpha )}\textbf{L} \bigl\Vert {\Xi }_{n-1}(t) \bigr\Vert + \frac{\alpha \textbf{L}}{ \mathsf{AB} (\alpha )\Gamma (\alpha )} \int _{0}^{t} (t-\varsigma )^{\alpha -1} \bigl\Vert {\Xi }_{n-1}(t) \bigr\Vert \,d\varsigma . \end{aligned}$$

Now, replacing $\Vert {\Xi }_{n-1}(t)\Vert $ by its value, we get

$$\begin{aligned} \bigl\Vert {\Xi }_{n}(t) \bigr\Vert \leq \biggl( \frac{1-\alpha }{ \mathsf{AB} (\alpha )}\textbf{L}+ \frac{\alpha \textbf{L}t^{\alpha }}{ \mathsf{AB} (\alpha )\Gamma (\alpha +1)} \biggr)^{2} \bigl\Vert {\Xi }_{n-2}(t) \bigr\Vert . \end{aligned}$$

Also, we have

$$\begin{aligned} \bigl\Vert {\Xi }_{n}(t) \bigr\Vert \leq \biggl( \frac{1-\alpha }{ \mathsf{AB} (\alpha )}\textbf{L}+ \frac{\alpha \textbf{L}t^{\alpha }}{ \mathsf{AB} (\alpha )\Gamma (\alpha +1)} \biggr)^{3} \bigl\Vert {\Xi }_{n-3}(t) \bigr\Vert . \end{aligned}$$

And finally,

20 $$\begin{aligned} \bigl\Vert {\Xi }_{n}(t) \bigr\Vert \leq & \biggl( \frac{1-\alpha }{ \mathsf{AB} (\alpha )}\textbf{L}+ \frac{\alpha \textbf{L}t^{\alpha }}{ \mathsf{AB} (\alpha )\Gamma (\alpha +1)} \biggr)^{n} \bigl\Vert {\Xi }_{0}(t) \bigr\Vert , \\ \leq & \biggl(\frac{1-\alpha }{ \mathsf{AB} (\alpha )}+ \frac{\alpha t^{\alpha }}{ \mathsf{AB} (\alpha )\Gamma (\alpha +1)} \biggr)^{n} \textbf{L}^{n} \max _{t\in [0,T]}{ \textbf{F}_{0}}(t). \end{aligned}$$

By choosing

21 $$\begin{aligned} {\textbf{F}} (t)=\sum_{i=0}^{n} {\Xi }_{i}(t), \end{aligned}$$

we are now in a position to define the following sequence for ${\textbf{F}} (t)$:

22 $$\begin{aligned} {\textbf{F}} (t)={\textbf{F}}_{n} (t)+\Theta _{n} (t), \end{aligned}$$

where $\Theta _{n} (t)\rightarrow 0$ when $n \rightarrow \infty $. Thus,

23 $$\begin{aligned} {\textbf{F}} (t)- {\textbf{F}_{n}}(t) =& \frac{1-\alpha }{ \mathsf{AB} (\alpha )} \textbf{N}\bigl({ \textbf{F}} (t)- \Theta _{n}(t)\bigr) \\ &{}+ \frac{\alpha }{ \mathsf{AB} (\alpha )\Gamma (\alpha )} \int _{0}^{t} (t- \varsigma )^{\alpha -1} \textbf{N}\bigl( {\textbf{F}} ( \varsigma )- { {\Theta }}_{n } {(\varsigma )}\bigr)\,d\varsigma . \end{aligned}$$

Now, we can write

$$\begin{aligned}& {\textbf{F}} (t)- {\textbf{F}_{0}}- \frac{1-\alpha }{ \mathsf{AB} (\alpha )} \textbf{N}\bigl({ \textbf{F}} (t)- \Theta _{n}(t)\bigr)- \frac{\alpha }{ \mathsf{AB} (\alpha )\Gamma (\alpha )} \int _{0}^{t} (t- \varsigma )^{\alpha -1} \textbf{N}\bigl( {\textbf{F}} ( \varsigma )- { {\Theta }}_{n } {(\varsigma )}\bigr)\,d\varsigma \\& \quad =\Theta _{n} (t)+\frac{1-\alpha }{ \mathsf{AB} (\alpha )} \bigl[ \mathcal{{ \textbf{N}}}\bigl({\textbf{F}} (t)- \Theta _{n}(t)\bigr)- \mathcal{{ \textbf{N}}}\bigl({\textbf{F}} (t) \bigr) \bigr] \\& \qquad {}- \frac{\alpha }{ \mathsf{AB} (\alpha )\Gamma (\alpha )} \int _{0}^{t} (t- \varsigma )^{\alpha -1} \bigl[\textbf{N}\bigl( {\textbf{F}} ( \varsigma )- { {\Theta }}_{n } {(\varsigma )}\bigr)-\textbf{N}\bigl( { \textbf{F}} (\varsigma ) \bigr) \bigr]\,d\varsigma . \end{aligned}$$

Taking the standard norm on both sides of the above equation, we conclude that

$$\begin{aligned}& \Vert {\textbf{F}} (t)- {\textbf{F}_{0}}(t)- \frac{1-\alpha }{ \mathsf{AB} (\alpha )} \textbf{N}\bigl({ \textbf{F}} (t)\bigr)+ \frac{\alpha }{ \mathsf{AB} (\alpha )\Gamma (\alpha )} \int _{0}^{t} (t-\varsigma )^{\alpha -1} \textbf{N}( { \textbf{F}} (\varsigma )\,d\varsigma \Vert \\& \quad \leq \bigl\Vert \Theta _{n} (t) \bigr\Vert + \frac{1-\alpha }{ \mathsf{AB} (\alpha )} \Vert \textbf{N}\bigl( { \textbf{F}} (\varsigma )- { {\Theta }}_{n } {(\varsigma )}\bigr)-{\textbf{N}}\bigl( { \textbf{F}} (\varsigma ) \bigr) \biggl\Vert \\& \qquad {} +\frac{\alpha }{ \mathsf{AB} (\alpha )\Gamma (\alpha )} \int _{0}^{t} (t-\varsigma )^{\alpha -1} \biggr\Vert \textbf{N}\bigl( {\textbf{F}} ( \varsigma )- { {\Theta }}_{n } {(\varsigma )}\bigr)-{\textbf{N}}\bigl( { \textbf{F}} ( \varsigma ) \bigr)\Vert \,d\varsigma \\& \quad \leq \bigl\Vert \Theta _{n} (t) \bigr\Vert + \frac{1-\alpha }{ \mathsf{AB} (\alpha )} \textbf{L} \bigl\Vert \Theta _{n-1} (t) \bigr\Vert + \frac{ \alpha t^{\alpha }}{ \mathsf{AB} (\alpha )\Gamma (\alpha +1)} \textbf{L} \bigl\Vert \Theta _{n-1} (t) \bigr\Vert . \end{aligned}$$

If $n\rightarrow \infty $, the right-hand side of the equation tends to zero. So, one gets

24 $$ \textbf{F}(t)- {\textbf{F}_{0}}= \frac{1-\alpha }{ \mathsf{AB} (\alpha )} \textbf{N}\bigl( \textbf{F}(t)\bigr)+ \frac{\alpha }{ \mathsf{AB} (\alpha )\Gamma (\alpha )} \int _{0}^{t} (t- \varsigma )^{\alpha -1} \textbf{N}\bigl( \textbf{F} {( \varsigma )}\bigr)\,d\varsigma . $$

And that was what we were trying to prove in this subsection.

### The uniqueness of the solution

In this part, we are looking for the proof of the uniqueness of the solution related to the model. To do this, let us consider that model admits two solutions ${\textbf{F}} (t)$ and ${\textbf{N}} (t)$. Then we can write

25 $$\begin{aligned} \bigl\Vert {\textbf{F}} (t)-{\textbf{N}} (t) \bigr\Vert \leq & \frac{1-\alpha }{ \mathsf{AB} (\alpha )}\textbf{L} \bigl\Vert {\textbf{F}} (t)-{ \textbf{N}} (t) \bigr\Vert + \frac{\alpha \textbf{L}t^{\alpha }}{ \mathsf{AB} (\alpha )\Gamma (\alpha +1)} \bigl\Vert {\textbf{F}} (t)-{\textbf{N}} (t) \bigr\Vert \\ \leq & \biggl(\frac{1-\alpha }{ \mathsf{AB} (\alpha )} \mathcal{\textbf{L}} + \frac{\alpha \mathcal{\textbf{L}} t^{\alpha }}{ \mathsf{AB} (1+\alpha )\Gamma (\alpha )} \biggr) \bigl\Vert {\textbf{F}} (t)-{\textbf{N}} (t) \bigr\Vert \end{aligned}$$

26 $$\begin{aligned} \vdots & \\ \end{aligned}$$

27 $$\begin{aligned} \leq & \biggl(\frac{1-\alpha }{ \mathsf{AB} (\alpha )} \mathcal{\textbf{L}} + \frac{\alpha \mathcal{\textbf{L}} t^{\alpha }}{ \mathsf{AB} (1+\alpha )\Gamma (\alpha )} \biggr)^{n} \bigl\Vert {\textbf{F}} (t)-{ \textbf{N}} (t) \bigr\Vert . \end{aligned}$$

If $\frac{1-\alpha }{ \mathsf{AB} (\alpha )} \mathcal{\textbf{L}} + \frac{\alpha \mathcal{\textbf{L}} t^{\alpha }}{ \mathsf{AB} (1+\alpha )\Gamma (\alpha )} <1$ holds, we get $\Vert {\textbf{F}} (t)-{\textbf{N}} (t)\Vert \leq 0$, from which the equality ${\textbf{F}} (t)={\textbf{N}} (t)$ results. This means that the system has a unique solution.

## A numerical method

In recent years, various approximate methods have been used to solve the system of fractional-order differential equations. In each of these methods, a specific idea is used to discretize the problem and approximate the solution of the system.

In order to express the method, we first need to consider a fractional delay differential equation given by

28 $$ \textstyle\begin{cases} {}^{\mathsf{C}} \mathcal{D} ^{\alpha }{\mathcal{R} }(t) = \mathcal{Q}(t, \mathcal{R}(t), \mathcal{R}(t-\tau _{1}), \mathcal{R}(t-\tau _{2})),& t \in [0,T], \\ \mathcal{R}(t)=\psi (t),& t\in [-\tau ,0]. \end{cases} $$

To determine the numerical method, we first consider the domain discretization as follows:

$$ t_{n} =n\hslash , \quad n= -k, -k+1,\ldots -1, 0, 1, \ldots N, \hslash := \frac{T}{N}=\frac{\tau }{k}. $$

Keeping these notations in mind, we obviously have $\mathcal{R}(t_{j})=\psi (t_{j})$, $j=-k, -k+1,\ldots , -1, 0 $.

In other words, it can be written

$$ \mathcal{R}(t_{j}- \tau ) = \mathcal{R}(jh- k\hslash ) = \mathcal{R}(t_{j-k}),\quad j = 0,1,\ldots ,N. $$

The main assumption of the numerical method is that we have approximated the value as the solution function in points

$$ \mathcal{R}(t_{j}), \quad j = -k, -k+1,\ldots -1, 0, 1, \ldots, n, $$

and now we are looking for the value of $\mathcal{R}(t_{n+1})$.

The next idea used is to apply the definition of the integral operator (6), which results in the following relation:

29 $$\begin{aligned} \mathcal{R}(t) - \mathcal{R}(t_{0}) =& \frac{1-\alpha }{{\mathsf{AB}} (\alpha )} \mathcal{R} (t )+ \frac{\alpha }{\Gamma (\alpha ){\mathsf{AB}} (\alpha )} \\ &{}\times \int _{t_{0}}^{t}(t- \varpi )^{\alpha -1} \mathcal{Q}\bigl(\varpi , \mathcal{R}(\varpi ), \mathcal{R}(\varpi -\tau _{1}), \mathcal{R}(\varpi -\tau _{2})\bigr)\,d\varpi . \end{aligned}$$

Setting $t=t_{n+1}$ in (29) gives

30 $$\begin{aligned}& \mathcal{R}(t_{n+1}) - \mathcal{R}(t_{0}) \\& \quad = \frac{1-\alpha }{{\mathsf{AB}} (\alpha )} \mathcal{R} (t_{n+1} )+\frac{\alpha }{\Gamma (\alpha ){\mathsf{AB}} (\alpha )} \\& \qquad {}\times \int _{t_{0}}^{t_{n+1}}(t_{n+1}- \varpi )^{\alpha -1} \mathcal{Q}\bigl(\varpi , \mathcal{R}(\varpi ), \mathcal{R}( \varpi -\tau _{1}), \mathcal{R}(\varpi -\tau _{2}) \bigr)\,d\varpi . \end{aligned}$$

Now, we utilize the product trapezoidal quadrature formula to approximate (30). Then, the corrector approximation for $\mathcal{R}(t_{n+1})$ is obtained as follows:

31 $$\begin{aligned} \mathcal{R}(t_{n+1}) =& \mathcal{R}(t_{0})+ \frac{1-\alpha }{{\mathsf{AB}} (\alpha )} \mathcal{R} (t_{n+1} ) \\ &{}+ \frac{\alpha \hslash ^{\alpha }}{{\mathsf{AB}} (\alpha ) \Gamma (\alpha +2)} \mathcal{Q}\bigl(t_{n+1}, \mathcal{R}(t_{n+1}), \mathcal{R}(t_{n+1}-\tau _{1}), \mathcal{R}(t_{n+1}-\tau _{2})\bigr) \\ &{}+\frac{\hslash ^{\alpha }}{ \Gamma (\alpha +2)}\sum_{j=0}^{n } \zeta _{j,n+1} \mathcal{Q}\bigl(t_{j}, \mathcal{R}(t_{j}), \mathcal{R}(t_{j}-\tau _{1}), \mathcal{R}(t_{j}- \tau _{2})\bigr) \\ =& \mathcal{R}(t_{0})+\frac{1-\alpha }{{\mathsf{AB}} (\alpha )} \mathcal{R}^{P} (t_{n+1} ) \\ &{}+ \frac{\alpha \hslash ^{\alpha }}{{\mathsf{AB}} (\alpha ) \Gamma (\alpha +2)} \mathcal{Q}\bigl(t_{n+1}, \mathcal{R}^{P}(t_{n+1}), \mathcal{R}(t_{n+1-k_{1}}), \mathcal{R}(t_{n+1-k_{2}})\bigr) \\ &{}+ \frac{ \alpha \hslash ^{\alpha }}{ {\mathsf{AB}} (\alpha )\Gamma (\alpha +2)} \sum_{j=0}^{n } \zeta _{j,n+1}\mathcal{Q}\bigl(t_{j}, \mathcal{R}(t_{j}), \mathcal{R}(t_{j-k_{1}}), \mathcal{R}(t_{j-k_{2}})\bigr), \end{aligned}$$

where $k_{1}=\operatorname{ceil}(\tau _{1}/\hslash )$, $k_{2}=\operatorname{ceil}(\tau _{2}/\hslash ) $, and

32 $$ \begin{aligned} \zeta _{j,n+1}&= \textstyle\begin{cases} n^{\alpha +1}- (n-\alpha ) (n+1 )^{\alpha },& j=0, \\ (n-j+2 ) ^{\alpha +1}- (n-j )^{\alpha +1} -2 (n-j+1 ) ^{\alpha +1},& 1 \leq j\leq n , \\ 1,& j=n+1. \end{cases}\displaystyle \end{aligned} $$

The unknown term $\mathcal{R}^{P}(t_{n+1})$ that appears in (31) is also calculated using the product rectangle rule as follows:

33 $$\begin{aligned} \mathcal{R}(t_{n+1}) =& \mathcal{R}(t_{0}) + \frac{1-\alpha }{{\mathsf{AB}} (\alpha )} \mathcal{R} (t_{n} ) \\ &{}+ \frac{\alpha \hslash ^{\alpha }}{{\mathsf{AB}} (\alpha ) \Gamma (\alpha +2)} \sum_{j=0}^{n } \zeta _{j,n+1}\mathcal{Q}\bigl(t_{j}, \mathcal{R}(t_{j}), \mathcal{R}(t_{j}-\tau _{1}), \mathcal{R}(t_{j}- \tau _{2})\bigr), \\ =& \mathcal{R}(t_{0}) +\frac{1-\alpha }{{\mathsf{AB}} (\alpha )} \mathcal{R} (t_{n } ) \\ &{}+ \frac{ \alpha \hslash ^{\alpha }}{{\mathsf{AB}} (\alpha ) \Gamma (\alpha +2)} \sum_{j=0}^{n } \zeta _{j,n+1}\mathcal{Q}\bigl(t_{j}, \mathcal{R}(t_{j}), \mathcal{R}(t_{j-k_{1}}), \mathcal{R}(t_{j-k_{2}})\bigr), \end{aligned}$$

where

34 $$ \begin{aligned} \zeta _{j,n+1}&= \frac{\hslash }{\Gamma (\alpha )} \bigl[ (n-j+1 ) ^{\alpha }- (n-j )^{\alpha } \bigr]. \end{aligned} $$

## Simulation results and discussion

In this section, the numerical simulations corresponding to the proposed model along with the attached approximate method are examined. In all graphs, the following values are used as the default of the constants, unless we intentionally change some of these values in the graphs ourselves. These changes are expressed in each graph.

35 $$ \begin{aligned} &\rho =0.3,\qquad b=0.3,\qquad \beta =1.4,\qquad \eta =0.1, \qquad \epsilon =0.1, \\ & \rho _{1}=0.5,\qquad \rho _{2}=0.1, \qquad \mu =0.0324,\qquad \gamma =(b-\mu )/200,\qquad \omega =0.1, \\ &\eta _{4}=0.1,\qquad \delta =0.05, \qquad \eta _{2}=0.1,\qquad \eta _{3}=0.2, \qquad \phi =0.5. \end{aligned} $$

**Figure 1:**
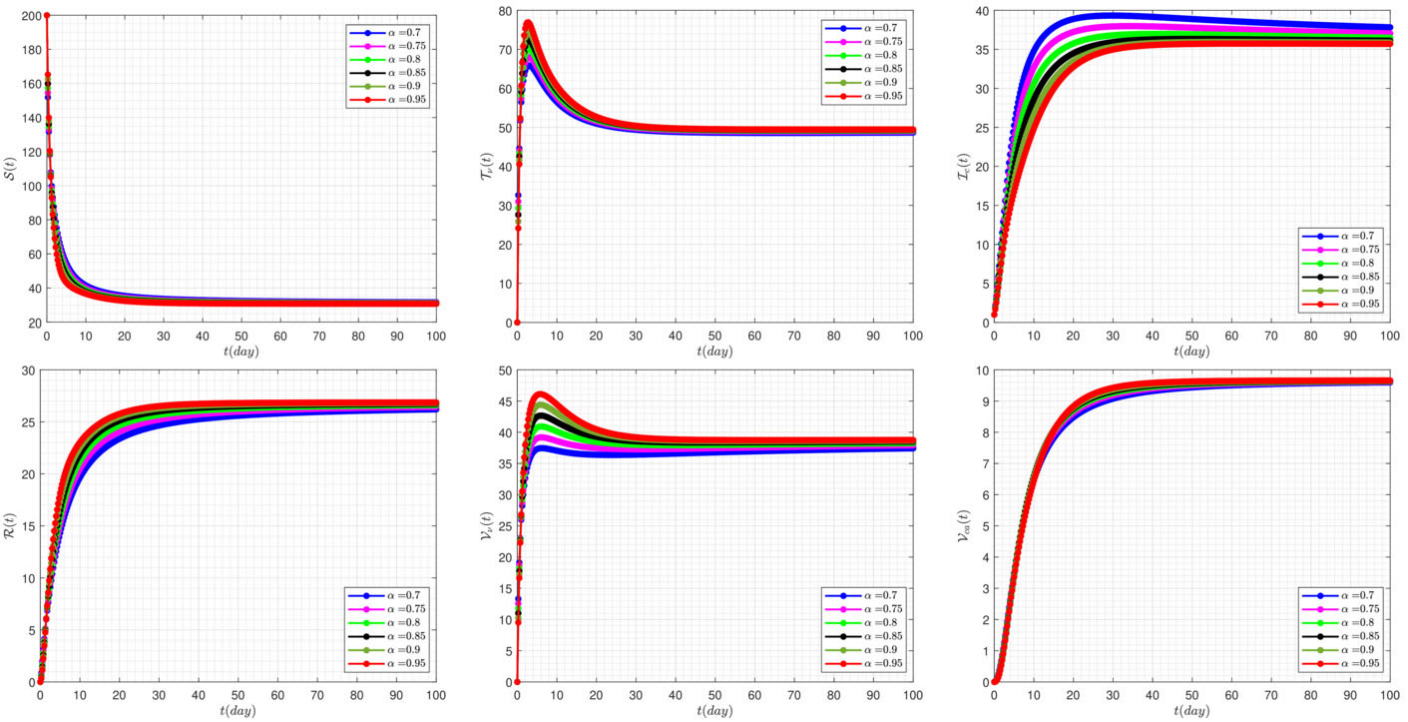
The impact of *α* on the results for $\tau _{1}=\tau _{2}=0$

The effect of fractional-order parameter (*α*) on the disease evolution, in the absence of delay parameters $\tau _{1}=\tau _{2}=0$, is shown in Fig. 1. It is seen that when *α* increases, then the susceptible and clinically infected classes decrease, and treated and vaccinated, recovered, vaccinated, and vaccinated carrier classes increase. In this case, all model solutions converge to the internal equilibrium point of the system.

**Figure 2:**
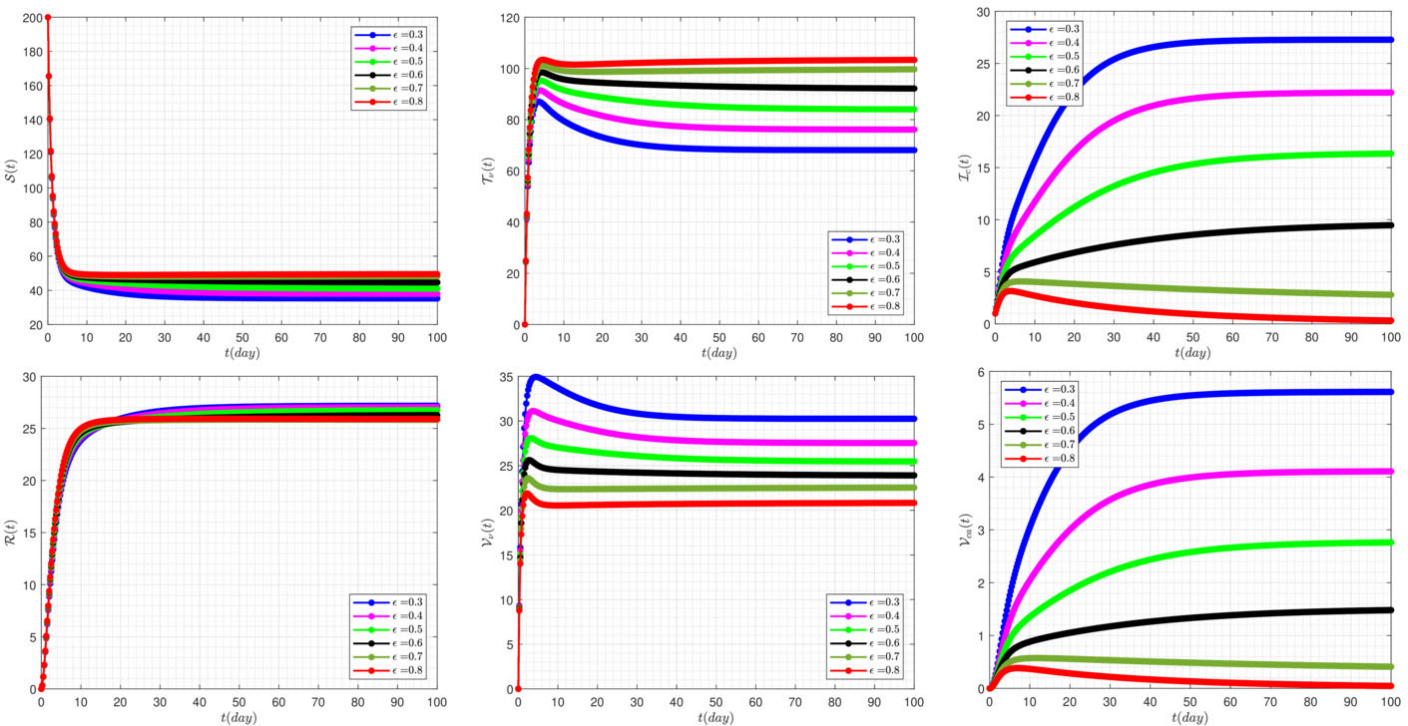
The impact of *ϵ* on the results for $\tau _{1}=\tau _{2}=0$ and $\alpha =0.95$

The effect of the treating of vaccinated people rate (*ϵ*) on the disease prevalence, in the absence of delay parameters $\tau _{1}=\tau _{2}=0$, is presented in Fig. 2. In some diagrams of this figure, it can be seen that a change in the value of the *ϵ* parameter can cause very significant changes in the overall condition of the system. In a way, by taking some values, the behavior of some solutions will be completely different from others in general. For example, by taking $\epsilon =0.8$, the graphs related to the two solutions clinically infected classes and vaccinated carrier classes take on a damping state and tend to zero.

**Figure 3:**
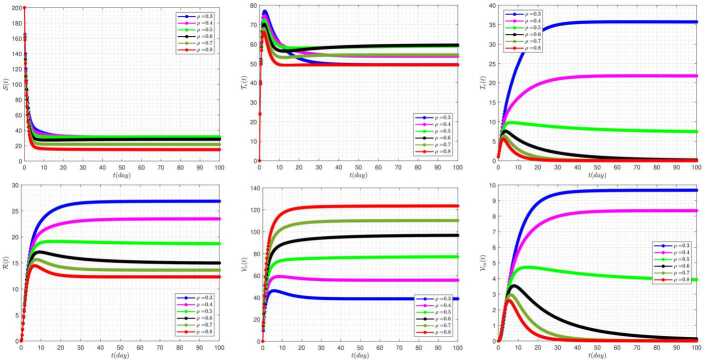
The impact of *ρ* on the results for $\tau _{1}=\tau _{2}=0$ and $\alpha =0.95$

Further, the effect of the rate of vaccination (*ρ*) on the disease evolution, in the absence of delay parameters $\tau _{1}=\tau _{2}=0$, is displayed in Fig. 3. In this figure it is clear that by taking $\rho =0.6, 0.7$, and 0.8 the graphs related to the two solutions clinically infected classes and vaccinated carrier classes take on a damping state and tend to zero. In other words, in these cases, the system moves to the disease-free equilibrium point. These conditions are fully consistent with the theoretical arguments presented in this paper and reference [42].

**Figure 4:**
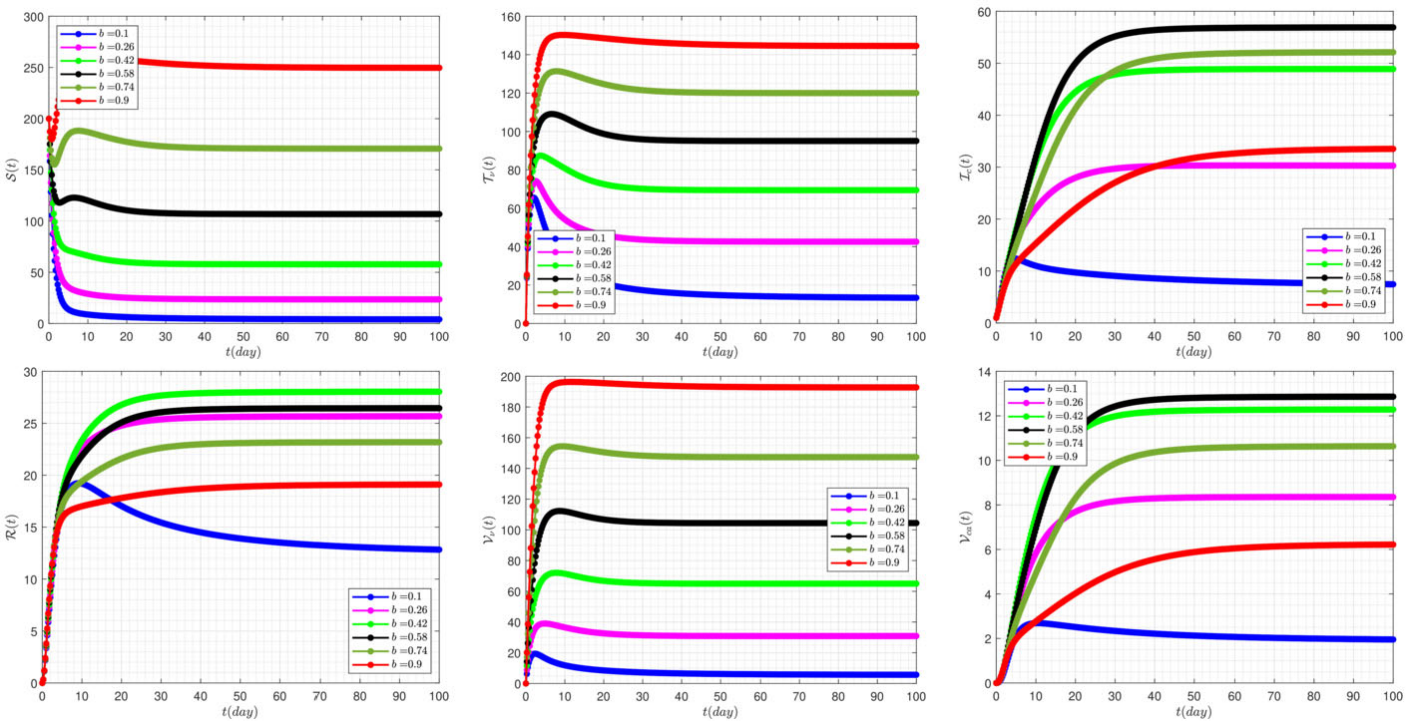
The impact of *b* on the results for $\tau _{1}=\tau _{2}=0$ and $\alpha =0.95$

The effect of the rate of vaccination (*b*) on the disease dynamics, in the absence of delay parameters $\tau _{1}=\tau _{2}=0$, has been plotted in Fig. 4. The diagrams in this figure clearly show that the curves corresponding to $b=0.1$ always create the lowest values in all possible solutions in the system. Taking the value stated in this case into account the system moves towards stability at the endemic equilibrium point $\mathcal{B}_{2}$.

**Figure 5:**
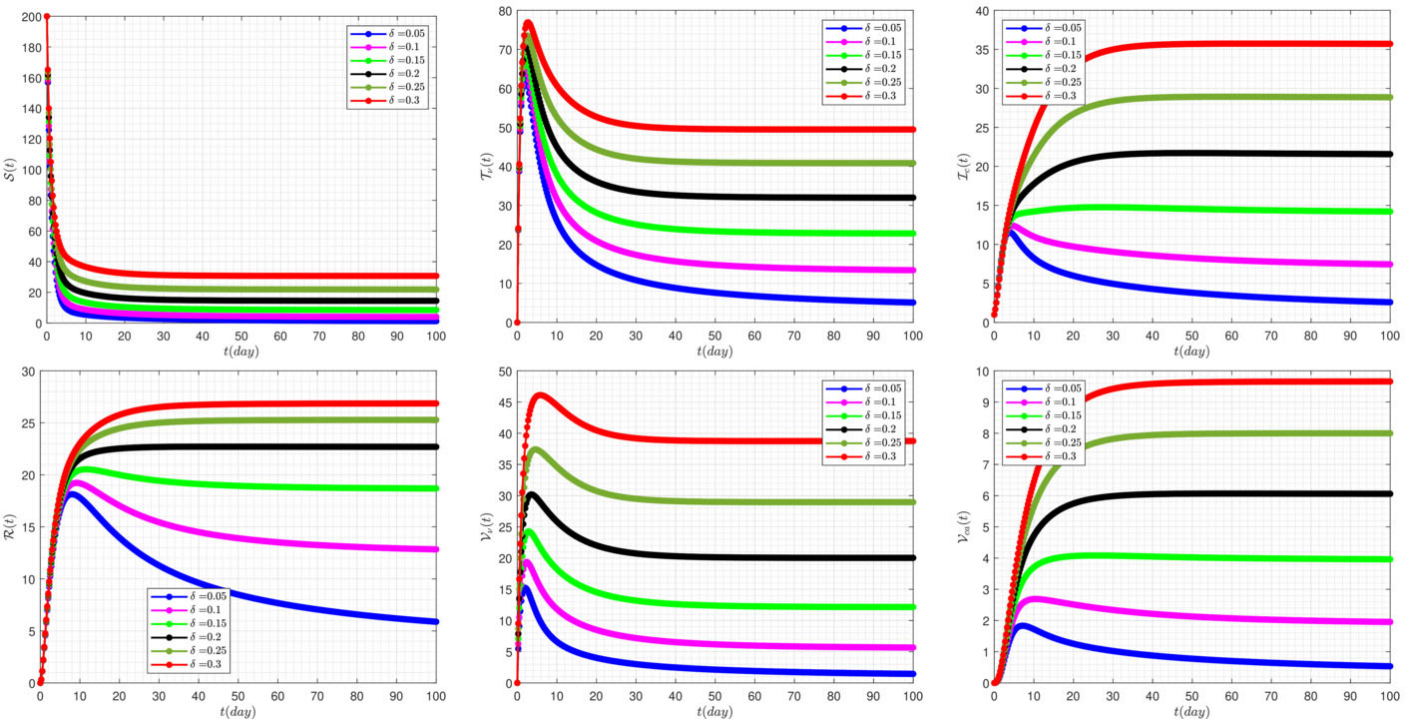
The impact of *δ* on the results for $\tau _{1}=\tau _{2}=0$ and $\alpha =0.95$

To investigate the significant effect of the rate of culling of clinical infective and vaccinated carrier people (*δ*) on the disease dynamics, in the absence of delay parameters $\tau _{1}=\tau _{2}=0$, we have presented Fig. 5. A distinctive feature of the diagrams in this figure is that the final values for system solutions always have an alignment correlation to the parameter *δ*. In other words, by increasing the value of parameter *δ*, the equilibrium points of the system are placed in higher levels.

**Figure 6:**
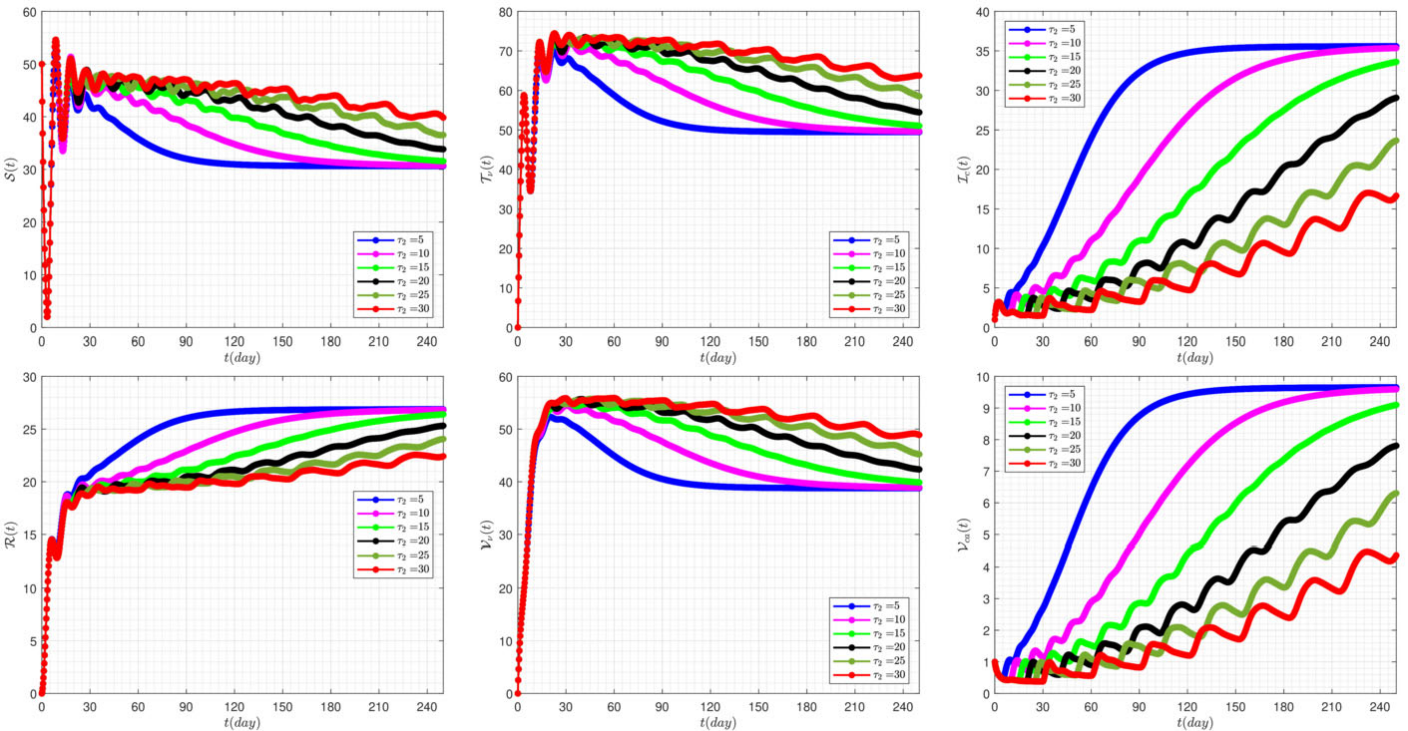
The impact of $\tau _{2}$ on the results for $\tau _{1}=3$ and $\alpha =0.95$

Figure 6 exhibits the effect of the rate of delay parameter ($\tau _{2}$) on the disease dynamics of the model when we take $\tau _{1}=3$. As we have seen in this figure, by increasing the delay parameter $\tau _{2}$, the solutions $\mathcal{S} (t)$, $\mathcal{T}_{\nu }$, $\mathcal{V}_{\nu } (t)$ have similar trends. The same is true also for $\mathcal{I}_{c} (t)$, $\mathcal{R}(t)$, $\mathcal{V}_{ca} (t)$. Also, by increasing the value of the parameter $\tau _{2}$, the system shows more oscillating behavior.

**Figure 7:**
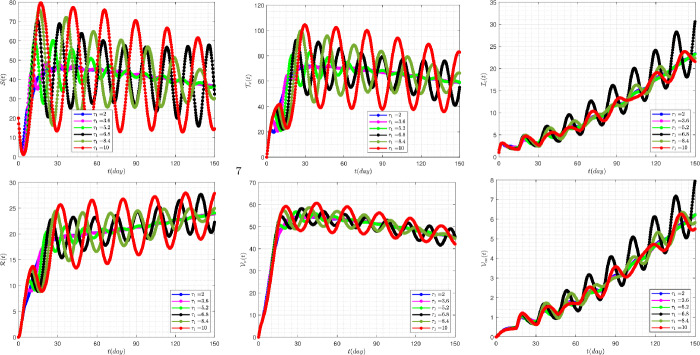
The impact of $\tau _{1}$ on the results for $\tau _{2}=15$ and $\alpha =0.95$

Figure 7 demonstrates the effect of the rate of delay parameter ($\tau _{1}$) on the disease variation of the model when we take $\tau _{2}=15$. The occurrence of oscillating properties in this figure is very obvious. In addition, by increasing the value of the delay parameter $\tau _{1}$, the system reveals more severe instability behavior.

**Figure 8:**
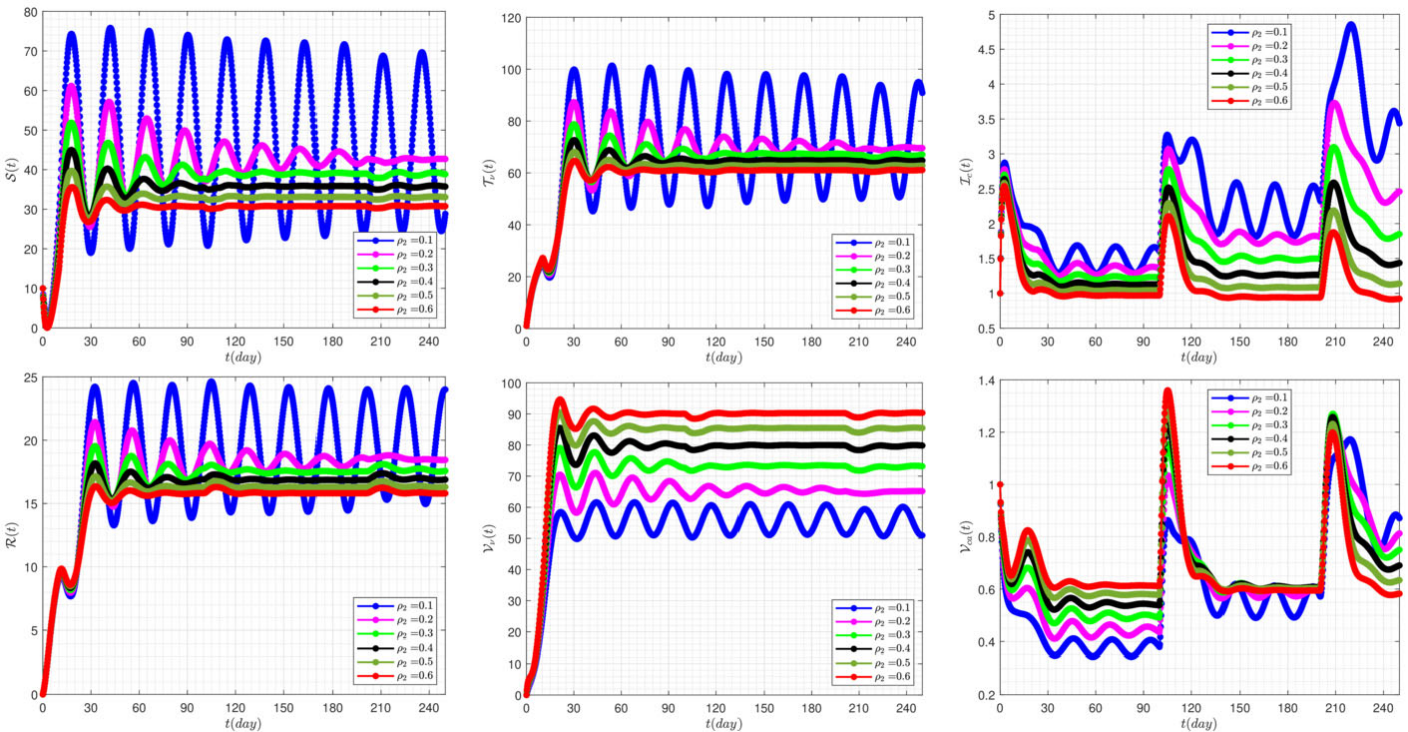
The impact of $\rho _{2}$ on the results for $\tau _{1}=10$, $\tau _{2}=100$, and $\alpha =0.95$

Figure 8 demonstrates the effect of the rate of vaccinating susceptible people ($\rho _{2}$) on the disease dynamics for delay parameters $\tau _{1}=10$, $\tau _{2}=100$. In this figure, the high sensitivity of the model’s response behavior to the parameter $\rho _{2}$ can be seen. For example, by considering $\rho _{2}=0.1$, the behavior of the system is quite volatile and unstable. While for the bigger values of this parameter, the system’s response show more stable behavior.

**Figure 9:**
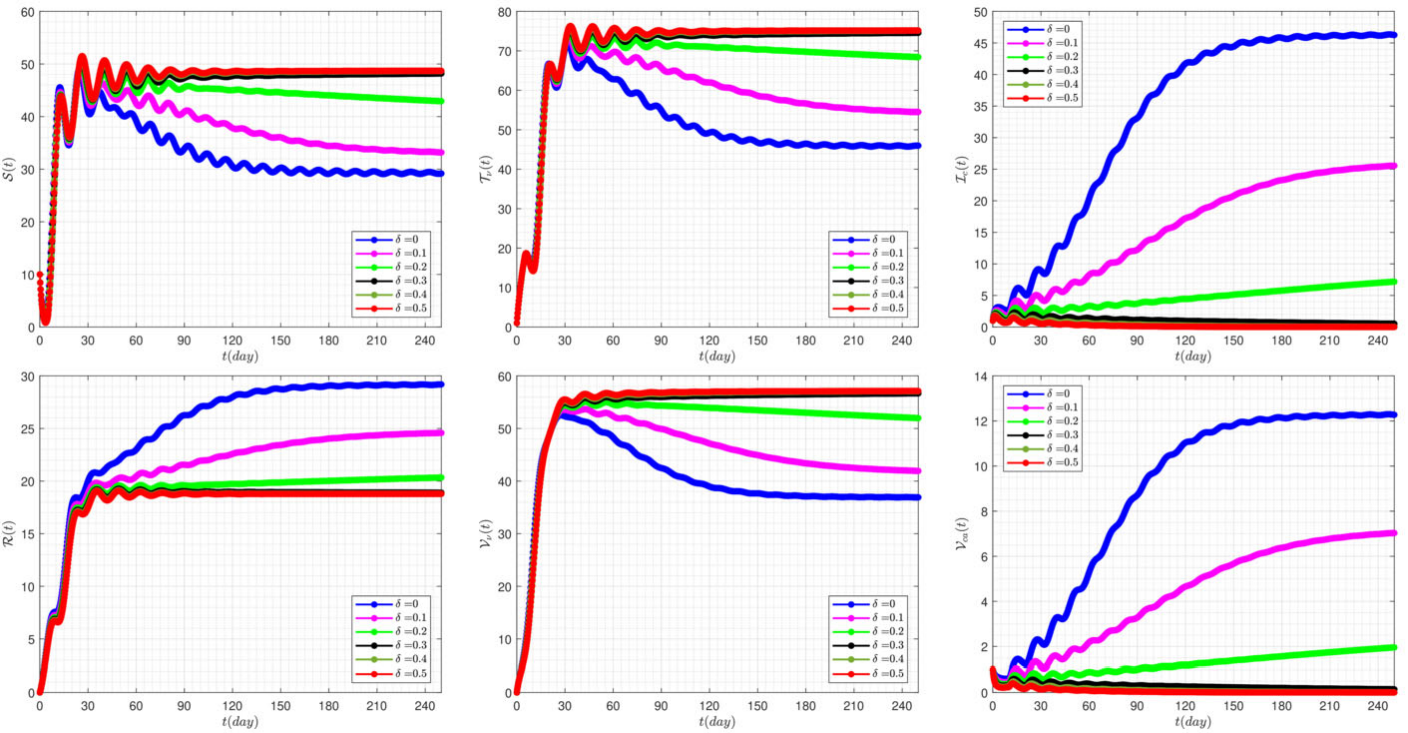
The impact of *δ* on the results for $\tau _{1}=5$, $\tau _{2}=10$, and $\alpha =0.95$

The effect of the rate of culling of clinical infective and vaccinated carrier people (*δ*) on the disease dynamics, for delay parameters $\tau _{1}=5$, $\tau _{2}=10$, has been displayed in Fig. 9.

**Figure 10:**
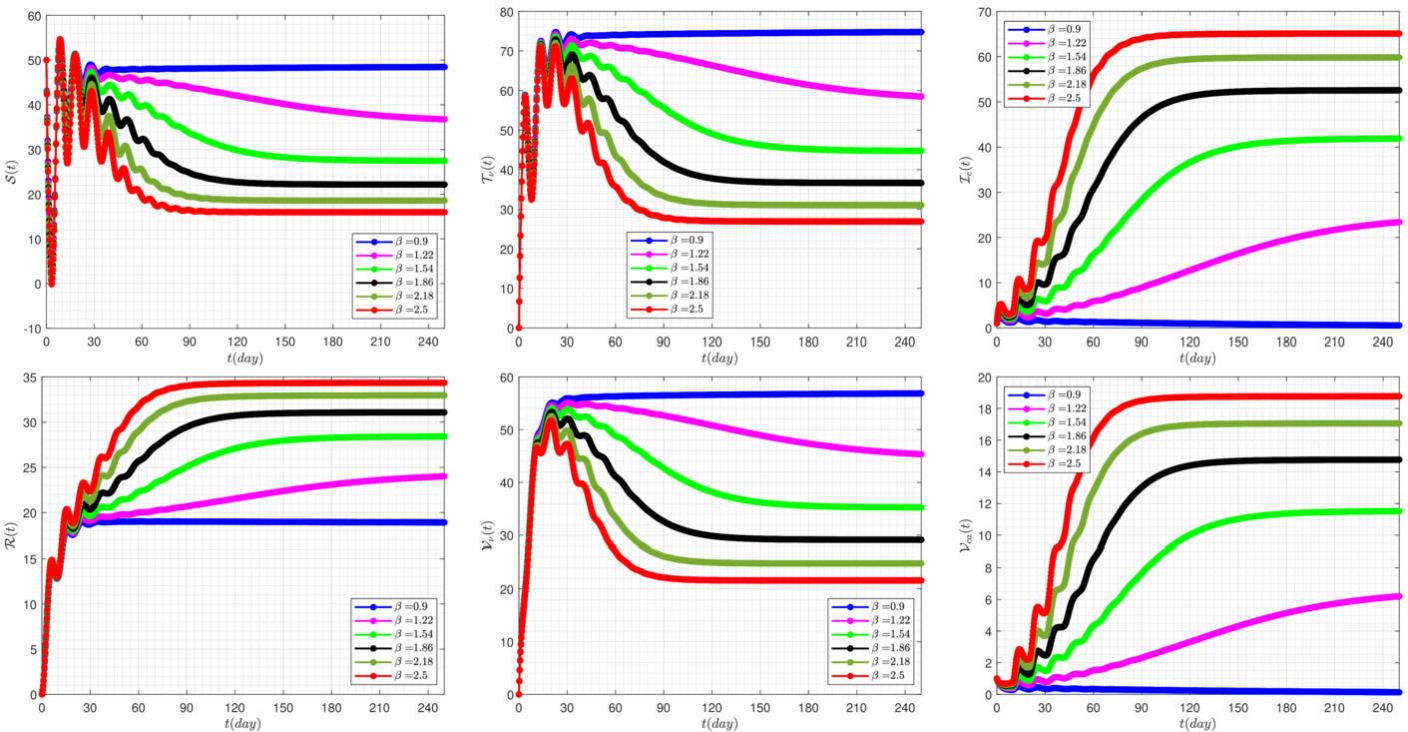
The impact of *β* on the results for $\tau _{1}=3$, $\tau _{2}=10$, and $\alpha =0.95$

The influence of the transmission rate (*β*) on the disease dynamics, for delay parameters $\tau _{1}=3$, $\tau _{2}=10$, is investigated in Fig. 10. It can be seen that for the $\beta =0.9$, the system tends toward the disease-free equilibrium $\mathcal{B}_{1}$, and for the other considered values to the endemic equilibrium point of $\mathcal{B}_{2}$. Therefore, *β* can play a very important and constructive role in the behavior and dynamics of the disease.

## Conclusion

Humans have been exposed to infectious diseases throughout history and have always tried to be able to control the spread of the disease and then find an effective treatment process to treat the disease. One way to control infectious diseases is to study the dynamic systems that describe the extent and prevalence of the disease. On the other hand, applying the modern concepts presented in the study of these dynamic systems can lead to dramatic advances in the study and control of this disease. In this article, we examined the prevalence of hand-foot-mouth disease in a certain population that has been described by a fractional system of ordinary differential equations. The model is constructed using the Atangana–Baleanu fractional derivative and two constant parameters to apply the time delay in the solutions. In order to take advantage of the concept of memory in the evolution of the model, we have employed a well-known derivative with fractional order as well as two delay parameters in the model.

Several numerical simulations revealed the effects of the fractional parameter, and the existence of two delay parameters in the disease dynamic. Through some performed experiments, we also investigated the sensitivity analysis of the model to some numerical parameters of the model. In some cases, it was observed that with a slight change in some of these parameters, very fundamental changes in the behavior of system responses occur. Another noteworthy aspect of this paper is the high degree of influence of the model on the delay parameters. For some specific values for the parameters in the system, unstable chaotic behavior can be observed in the system responses. The results of the model presented in this paper provide the ability to investigate the effects of time delay parameters, prophylactic vaccination, reactive vaccination, prophylactic treatment, and reaction-reflecting parameters on disease outbreak in a population. The process presented in this article can be applied to other infectious disease models.
